# Automated Deep Learning Quantification of Avascular Area and Intravitreal Neovascularization in Retinal Flatmounts of Rodent Oxygen-Induced Retinopathy Models

**DOI:** 10.1167/tvst.15.6.41

**Published:** 2026-06-30

**Authors:** Neal S. Shah, Aniket Ramshekar, Bright Asare-Bediako, Morgan P. Tankersley, Heng-Chiao Huang, Shreya Beri, Eric Kunz, Aaron Y. Lee, M. Elizabeth Hartnett

**Affiliations:** 1 Byers Eye Institute Department of Ophthalmology, Stanford University School of Medicine, Stanford, CA, USA; 2 Department of Ophthalmology, Chang Gung Memorial Hospital, Chiayi, Taiwan; 3 John A. Moran Eye Center, University of Utah, Salt Lake City, UT, USA; 4 John F. Hardesty Department of Ophthalmology and Visual Sciences, Washington University in St. Louis, St. Louis, MO, USA

**Keywords:** retinopathy of prematurity, deep learning model, oxygen-induced retinopathy, intravitreal neovascularization, avascular area

## Abstract

**Purpose:**

To develop a single deep learning model that quantifies the retinal avascular area (AVA) and intravitreal neovascularization (IVNV) in rodent oxygen-induced retinopathy (OIR) models.

**Methods:**

A U-Net–based model was developed to analyze AVA and IVNV in lectin-stained retinal flatmounts. The model was trained on 325 images (267 mouse and 58 rat) and evaluated on an independent test set of 37 images (18 mouse and 19 rat) annotated by human graders. We assessed intergrader reliability and agreement at metric and pixel levels. Mouse pixel-level performance was also compared with a previously published model.

**Results:**

Intergrader reliability was high for percent AVA (mouse intraclass correlation coefficient [ICC] = 0.840; rat ICC = 0.971), moderate for rat percent IVNV (ICC = 0.509), and low for mouse percent IVNV (ICC = −0.082). Metric-level correlation was strong in rat OIR (percent AVA *r* = 0.979; percent IVNV *r* = 0.943) and for mouse percent AVA (*r* = 0.957), but weak for mouse percent IVNV (*r* = 0.265). The Dice similarity coefficient was high for total retina (TR)/AVA and moderate for IVNV (rat: TR = 0.983, AVA = 0.924, IVNV = 0.612; mouse: TR = 0.975, AVA = 0.912, IVNV = 0.601). In mouse OIR, the Dice similarity coefficient matched or exceeded the previously published model (AVA = 0.912 vs. 0.887; IVNV = 0.601 vs. 0.559). Reviewers selected the IVNV mask created by the model in 83.3% of qualitative comparisons.

**Conclusions:**

Our deep learning model supports automated rat OIR analysis while maintaining mouse performance and may improve reproducibility of OIR measurements.

**Translational Relevance:**

Rodent OIR models are necessary to understand retinopathy of prematurity (ROP) pathophysiology. Our deep learning model effectively quantifies features of ROP recapitulated by both mouse and rat OIR.

## Introduction

Retinopathy of prematurity (ROP) remains a leading cause of childhood blindness worldwide despite advances in neonatal care, underscoring the need to understand the evolving pathophysiology.^1^^–^^3^ Because of the inability to test mechanisms of disease on infant eyes, preclinical studies using animal models that mimic features of vascular pathology seen in ROP are important. Rodent oxygen-induced retinopathy (OIR) models are widely used to evaluate human ROP. The mouse and rat models test different features of human ROP and are both necessary to understand the pathophysiology of the disease. In a widely used mouse OIR model,^4^ pups are exposed to 75% oxygen from postnatal day 7 to 12. The exposure to high oxygen causes central vaso-obliteration of already developed capillaries (i.e., retinal avascular area [AVA]). After return to room air, intravitreal neovascularization (IVNV) develops at the vascular–avascular borders and is commonly assessed at postnatal day 17.^5^^,^^6^ In the widely used rat 50/10 OIR,^7^ newborn pups with minimal retinal vascular development are exposed to oxygen fluctuations between 50% and 10% every 24 hours from postnatal day 0 to 14. Following oxygen fluctuations, pups are placed in room air. There is a peripheral region of avascular retina that allows the study of ongoing developing angiogenesis to the ora serrata and IVNV at the junction of the vascular–avascular border, commonly measured at postnatal day 18 to 20. For both models, retinas are dissected from fixed enucleated eyes, stained to label blood vessels (typically with isolectin-B4), flatmounted for fluorescence microscopy, and scored by at least two masked human graders to manually quantify avascular retinal area, capillary density, and IVNV as a percent of total retinal area.

Flatmount scoring by human graders is time consuming and demonstrates substantial intergrader and intragrader variability. IVNV is especially challenging to score consistently due to highly variable three-dimensional morphologies that are difficult to distinguish from normal vasculature in two-dimensional fluorescence images. These challenges have motivated the development of computational approaches to improve quantification. Several groups have developed automated or semi-automated methods to reduce grader burden and improve reproducibility, including computer-aided quantification approaches,^6^ machine learning pipelines that quantify tufts and avascular regions,^8^ and automated measurement of peripheral avascular area in rat OIR.^9^ More recently, deep learning approaches using convolutional neural networks have been applied to OIR image analysis,^10^ and open-source murine OIR flatmount datasets have been released to support larger scale and more generalizable model development.^11^ Despite these advances, existing computational methods that have been developed are validated primarily on images from single institutions or require user-defined parameters for scoring, which may limit their generalizability across different imaging protocols, sample preparation techniques, and laboratory conditions. Additionally, most tools focus on either mouse or rat models separately, requiring researchers to use different analysis pipelines based on the species studied. However, addressing different hypotheses requires the use of both animal models. Therefore, there is a need for a tool that quantifies both AVA and IVNV in mouse and rat OIR within a single framework and is tested on images collected from multiple sources to improve application to samples from different laboratories and imaging conditions. In this study, we postulated that a single deep learning model could extend automated OIR quantification to rat retinal flatmounts while maintaining mouse OIR performance comparable to a previously published deep learning model.^10^ Our goal was to develop a generalizable, efficient, and reproducible tool that can be widely adopted to standardize OIR quantification across laboratories and models.

## Methods

### Study Overview

This study was conducted in three sequential phases: image dataset assembly, deep learning model development, and independent evaluation against human graders. We first assembled annotated retinal flatmount images for model training (Supplementary Fig. S1). We then trained a deep learning model to annotate total retina (TR), AVA, and IVNV. Finally, we evaluated the trained deep learning model on an independent test set of retinal flatmount images and compared its performance with that of human graders as the reference.

### Image Dataset Assembly

#### Image Datasets and Annotation Sources

We used three image datasets for model development and evaluation. First, we collected 72 rat retinal flatmount images from our laboratory that had expert annotation masks for IVNV only. These images were used in an intermediate stage of model development focused specifically on rat IVNV. Second, we assembled a 325-image dataset for final model development. This dataset included 107 retinal flatmount images annotated by expert human graders in our laboratory, including 49 mouse images and 58 rat images, as well as 218 mouse retinal flatmount images selected from an open-source repository.^11^ For the open-source mouse images, candidate annotation masks for TR, AVA, and IVNV were initially generated using a previously published deep learning model.^10^ These candidate masks were used only as a starting point to accelerate annotation and were not automatically accepted for training. Instead, two experienced graders independently reviewed each candidate mask at the pixel level. Images were included in the training dataset only when both graders agreed that the annotation accurately represented the vascular structures and did not require substantial correction. Images with annotation errors or ambiguous vascular boundaries were excluded rather than corrected to avoid introducing inconsistent annotations. This curation process ensured that training images were included only when candidate masks closely matched expert interpretation of the vascular structures. Third, we assembled an independent test set consisting of 37 retinal flatmount images from a separate study that was not used for training, validation, or model selection. This test set included 18 mouse images and 19 rat images.

#### Image Preprocessing

Before training our deep learning model, we created a script to convert retinal flatmount images to grayscale, resized to 512 × 512 pixels, normalized for image intensity by rescaling to a standard 8-bit intensity range (0, 255), and linearly mapped to a fixed range (−1, +1). These steps were used to standardize image appearance across datasets. During training, image augmentation procedures were incorporated to improve robustness to variation in image appearance, including random horizontal and vertical flips, rotations, brightness and contrast adjustment, and noise injection.^12^

### Deep Learning Model Development

#### Training and Validation Set Construction

**Figure 1.**
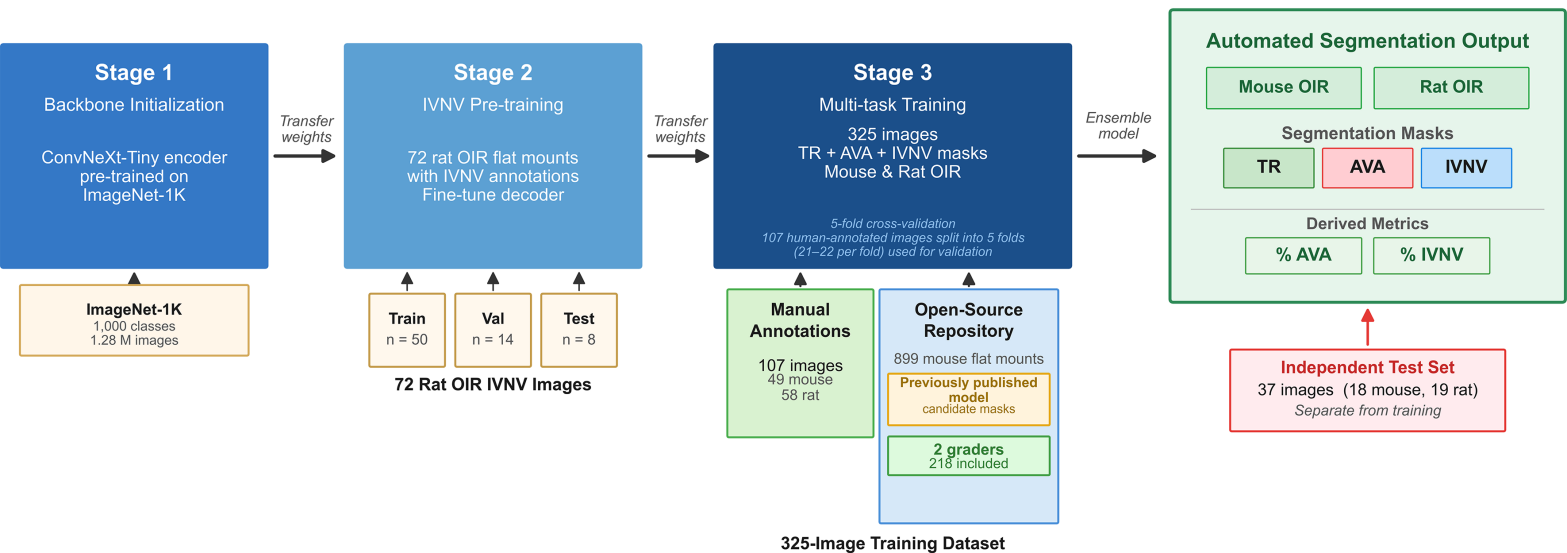
Three-stage progressive training framework for multi-task retinal flatmount annotation. In Stage 1 (backbone initialization), our deep learning model is initialized with ConvNeXt-Tiny weights pretrained on ImageNet-1K (1000 classes and 1.28 million images). In Stage 2 (IVNV pretraining), the model is fine-tuned on 72 rat OIR flatmount images annotated for IVNV. Training set (Train), *n* = 50; validation set (Val), *n* = 14; test set (Test), *n* = 8. In Stage 3 (multi-task training), multi-task training is performed on 325 images for simultaneous annotation of TR, AVA, and IVNV in mouse and rat OIR. The 325-image dataset includes 107 manually annotated images (49 from mouse and 58 from rat) and 218 mouse images curated from an open-source repository (899 total mouse retinal flatmount images), in which candidate masks were generated by a previously published model and reviewed by two graders. A five-fold cross-validation strategy used 107 human-annotated images split into five folds for validation. The final ensemble model was then applied to an independent test set of 37 images (automated segmentation output, *n* = 18 mouse retinal flatmount images and *n* = 19 rat retinal flatmount images).

We developed the model using a three-stage progressive training strategy (Fig. 1). In Stage 1, the model encoder was initialized using weights from ConvNeXt-Tiny pretrained on ImageNet-1K.^13^^,^^14^ In practical terms, this allowed the model to begin with previously learned general visual features, such as edges, textures, and shapes, before being adapted to retinal flatmount images. In Stage 2, weights from Stage 1 were used to train a rat IVNV-specific model with the 72 rat images that had IVNV-only annotations. This dataset was divided into training, validation, and test subsets of 50, 14, and 8 images, respectively. Stage 2 was trained for 40 iterations, and the checkpoint at iteration 30 was selected due to it achieving the best validation performance on rat IVNV. In Stage 3, weights from Stage 2 were transferred to the final model, which was then trained to simultaneously annotate TR, AVA, and IVNV using the 325-image dataset.

For final model development, we used a modified five-fold cross-validation approach.^15^ The 107 human-annotated images were partitioned into five folds, with 21 to 22 images per fold. In each fold, one partition served as the validation set and the remaining images were used for training together with the reviewed open-source mouse images. Depending on fold size, 303 to 304 images were used for training and 21 to 22 images were used for validation. Multi-task training was run for 120 iterations. The final model uses a five-fold ensemble, where predictions from all five folds are averaged to produce a single final output.

#### Deep Learning Model Architecture

We developed a deep learning model to annotate three regions from each retinal flatmount image: TR, AVA, and IVNV. The model was based on a modified U-Net architecture with attention mechanisms.^16^ U-Net is a commonly used neural network design for image annotation because it combines broad image context with fine spatial detail.^17^ The model used an encoder–decoder structure. The encoder extracted image features at multiple levels of detail, and the decoder used these features to reconstruct full-resolution annotation masks. Attention mechanisms were incorporated to help the model focus more strongly on image regions most relevant to each annotation task. The deep learning model outputs separate pixel-level maps for TR, AVA, and IVNV. The final model contained approximately 8.7 million trainable parameters. The encoder backbone was based on ConvNeXt-Tiny,^14^ which was chosen because of its strong performance in image-recognition tasks and compatibility with transfer learning.

#### Automated Scoring and Post-Processing

For automated scoring, images were preprocessed in the same manner as during model training. To improve prediction stability, an ensemble of five cross-validation models analyzed augmented versions of each image; specifically, each image was evaluated at 0°, 90°, 180°, and 270°, and each rotation was also mirrored, with predictions averaged across all variants and models to make the final segmentation less sensitive to image orientation and more robust (dihedral group 4 [D4] test-time augmentation). For each region, the model first produced a pixel-level confidence map showing how likely each pixel was to belong to TR, AVA, or IVNV. These confidence maps were then averaged across image augmentations and across ensemble models.

The averaged confidence maps were converted into final binary annotation masks using separate cutoffs for TR, AVA, and IVNV that were determined from the validation images and aggregated across folds. Predicted annotation masks were then scaled back to the original image resolution using nearest-neighbor interpolation.^18^ To improve annotation accuracy, AVA and IVNV masks were constrained to lie within the predicted TR mask. Isolated components smaller than 50 pixels were removed during post-processing. Final annotation masks were used to calculate percent areas of either AVA or IVNV. Percent AVA was defined as 100 × (AVA area/TR area), and percent IVNV was defined as 100 × (IVNV area/TR area).

### Independent Evaluation Against Human Graders

#### Human Grading of the Independent Test Set

Three masked human graders analyzed all images in the independent test set using Fiji (ImageJ 1.54p; National Institutes of Health, Bethesda, MD).^19^ The graders included one experienced grader and two less experienced graders based on previous retinal flatmount scoring experience. The experienced grader was identified before analysis and was used as the reference for secondary analyses because IVNV grading is known to have substantial intergrader variability.^8^^,^^10^ Definitions and representative example images of annotated retinal flatmounts from mouse and rat OIR were reviewed to standardize grading.

Each grader measured percent AVA and percent IVNV as previously described.^20^^–^^22^ For metric-level analysis, which refers to comparison of raw values (i.e., percent AVA and percent IVNV), we averaged percent AVA and percent IVNV across the three human graders to obtain a single value for each image, which we then compared with the corresponding value generated by our deep learning model.

Each human grader also saved their annotation masks for TR, AVA, and IVNV. For pixel-level analysis, which refers to comparison of the annotated regions at the pixel level, we generated a consensus mask for each region, such that a pixel was included when at least two of the three graders labeled that pixel as belonging to that region. We then compared these consensus masks with the corresponding masks generated by our deep learning model.

To compare efficiency, we recorded the time required for each grader to annotate each image. Graders started a timer at the beginning of scoring a retinal flatmount and stopped timing upon completion of annotation, with the timer paused during any intermittent breaks. For the deep learning model, we recorded total automated scoring time per image, including image preprocessing, model scoring, and post-processing.

#### Qualitative Review of Mouse IVNV Annotation Masks

Because mouse IVNV is particularly difficult to annotate consistently on retinal flatmount images, we performed a secondary qualitative analysis focused on the 18 mouse images in the independent test set. For each image, IVNV annotation masks from the deep learning model and from the three human graders were displayed side by side in a randomized, de-identified four-panel layout, with the original retinal flatmount image available as a reference. Three masked expert reviewers who had not participated in annotation and had not previously seen these images independently selected the panel they judged to be the most accurate IVNV annotation mask. Selections were recorded without discussion among reviewers.

### Statistical Analysis

Outcome measures were selected following the Metrics Reloaded framework,^23^ which recommended the Dice similarity coefficient for evaluation of semantic segmentation. For pixel-level analysis, agreement between model-generated annotation masks and human consensus annotation masks was assessed using the Dice similarity coefficient. For secondary pixel-level comparisons between our deep learning model and the previously published model, intersection over union (IoU) was also calculated.

For metric-level analysis, agreement between model-derived and human-derived percent AVA and percent IVNV values was evaluated using Pearson correlation and Spearman rank correlation. Bland–Altman analysis was used to assess systematic bias and 95% limits of agreement. Proportional bias was assessed by linear regression of the difference on the mean. Human intergrader reliability for percent AVA and percent IVNV was summarized using the intraclass correlation coefficient (ICC), based on a two-way random-effects model with absolute agreement, ICC(2,1). Data are summarized with 95% confidence intervals (CIs) for median Dice scores and for paired median differences across images, including comparisons between our deep learning model and the previously published model where applicable. Statistical significance was defined as *P* ≤ 0.05. No adjustment for multiple comparisons was performed.

### Libraries and Reproducibility

All analyses were conducted using a reproducible Python-based pipeline that regenerates tables, figures, overlays, and summary outputs from the endpoint tables and annotation masks. The model was implemented in PyTorch 2.8.0^24^ with supporting libraries including torchvision 0.23.0, Albumentations 2.0.8,^12^ NumPy 2.0.2, OpenCV 4.13.0.92, scikit-image 0.24.0,^25^ pandas 2.3.3, and Matplotlib 3.9.4.

### Software Repository

The model has been uploaded to the MONAI Model Zoo under the name oir_flatmount_segmentation. The model is also freely available with instructions provided via GitHub (https://github.com/hartnettlabteam/oir_flatmount_segmentation_app).

## Results

### Previously Published Deep Learning Model Was Unable to Quantify Rat OIR Retinal Flatmounts

The previously published deep learning model^10^ was not designed for rat OIR flatmounts; however, we applied it to the rat images as a baseline comparison. When applied to rat OIR retinal flatmount images, the model either failed to generate segmentation outputs or produced masks that were not visually accurate when looking at the original flatmount image (Supplementary Fig. S2). These findings highlight the need for a deep learning model that can quantify and annotate retinal flatmounts from both mouse and rat OIR models.

### Training and Validation of a Deep Learning Model for Simultaneous TR, AVA, and IVNV Annotation of Retinal Flatmounts From Mouse and Rat OIR Models

The average best validation Dice score across the five folds was 0.825 (SD = 0.018), with fold-specific values of 0.803, 0.831, 0.825, 0.852, and 0.816. In the best-performing fold, the final validation Dice scores were 0.981 for TR, 0.545 for IVNV, and 0.932 for AVA. For final use, we combined predictions from all five folds to produce one final output, and we applied region-specific cutoffs based on validation data (Fig. 1). Together, these findings show that our deep learning model could be trained to generate annotation masks for TR, AVA, and IVNV in retinal flatmounts from mouse and rat OIR models with high validation performance before evaluation on the independent test set.

### Human Intergrader Reliability Is High for Percent AVA in Mouse and Rat OIR Models But Low for Percent IVNV in Mouse OIR Model

**Figure 2.**
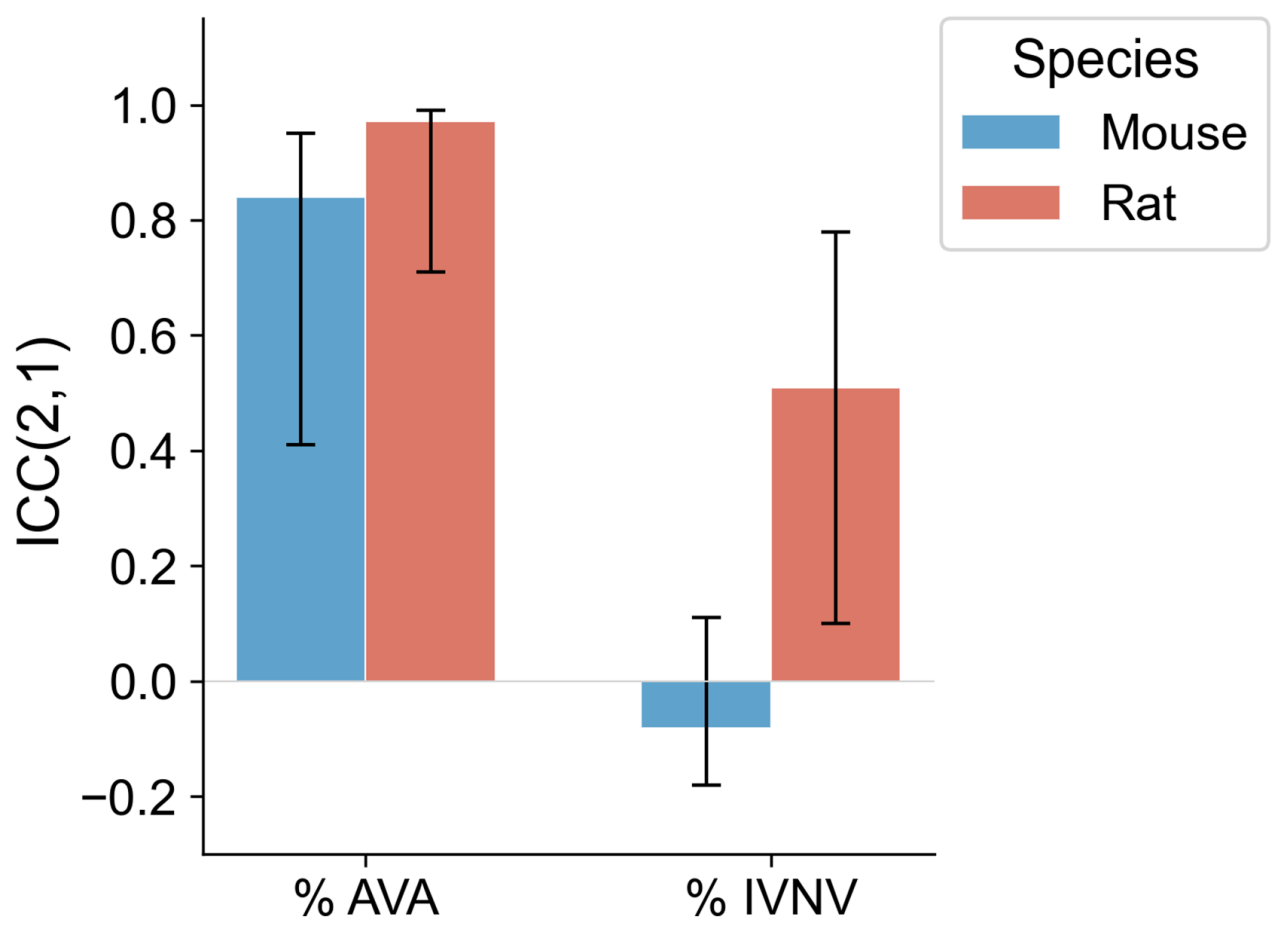
Intergrader reliability for percent AVA and percent IVNV in mouse and rat OIR retinal flatmounts. Intergrader reliability for mouse percent AVA: ICC(2,1) = 0.840, *P* = 3.39 × 10^−^^18^ (*n* = 18); for rat percent AVA: ICC(2,1) = 0.971, *P* = 1.02 × 10^−^^37^(*n* = 19); for mouse percent IVNV: ICC(2,1) = −0.082, *P* = 0.901 (*n* = 18); for rat percent IVNV: ICC(2,1) = 0.509, *P* = 4.45 × 10^−^^8^ (*n* = 19). *Bars* show ICC(2,1), and *error bars* show 95% CIs.

Before evaluating the performance of our deep learning model against our human graders, we first quantified agreement among our human graders. Using the independent test set (18 mouse images and 19 rat images), three human graders independently annotated each retinal flatmount image to generate percent AVA and IVNV for metric-level analysis along with annotation masks for pixel-level analysis. We assessed metric-level agreement among graders using ICCs. For mouse percent AVA, intergrader reliability was high (ICC = 0.840, *p* = 3.39 × 10^–18^, *n* = 18), whereas mouse percent IVNV showed poor agreement (ICC = –0.082, *P* = 0.901, *n* = 18). For rat endpoints, intergrader reliability was very high for percent AVA (ICC = 0.971, *P* = 1.02 × 10^–37^, *n* = 19) and moderate for percent IVNV (ICC = 0.509, *P* = 4.45 × 10^–8^, *n* = 19) (Fig. 2).

**Figure 3.**
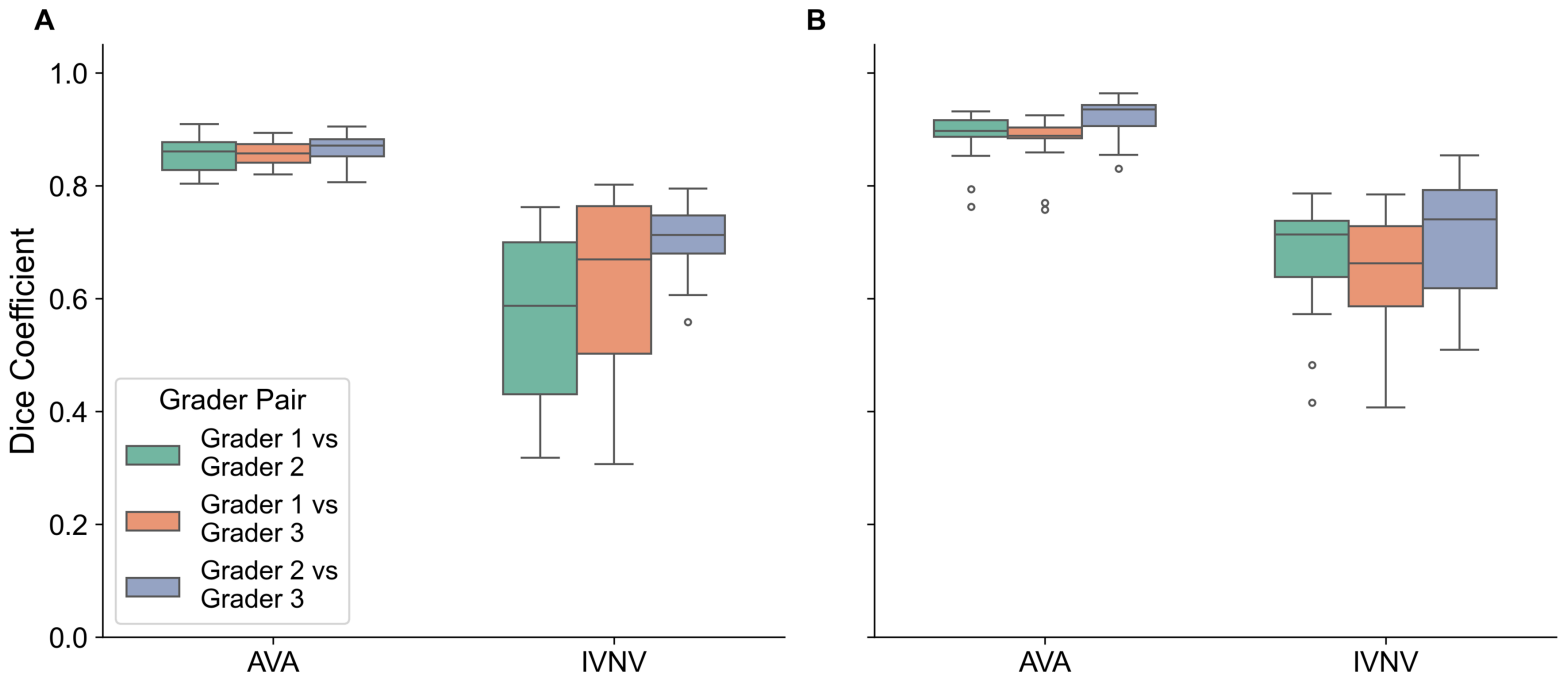
Intergrader pairwise Dice scores for AVA and IVNV mask annotations in mouse and rat OIR retinal flatmounts. Boxplots demonstrating distribution of pairwise Dice scores among all three grader pairs: (**A**) Mouse (*n* = 18), with median pairwise Dice coefficients of 0.860 for mouse AVA and 0.669 for mouse IVNV. (**B**) Rat (*n* = 19) images with median pairwise Dice coefficients of 0.897 for rat AVA and 0.713 for rat IVNV.

We also assessed pixel-level agreement among our human graders using pairwise Dice scores for AVA masks and IVNV masks. Agreement was higher for AVA than IVNV masks in both species (rat AVA: median pairwise Dice = 0.897, interquartile range [IQR] = 0.893–0.916; rat IVNV: median pairwise Dice = 0.713, IQR = 0.688–0.727; mouse AVA: median pairwise Dice = 0.860, IQR = 0.859–0.866; mouse IVNV: median pairwise Dice = 0.669, IQR = 0.628–0.691) (Fig. 3). Although graders often overlapped on IVNV location at the pixel level (reflected in mask Dice scores), their measurements of how much total IVNV was present differed substantially, leading to low ICCs. Taken together, these findings highlight the need for a deep learning model to standardize scoring across retinal flatmounts in mouse and rat OIR models.

### Metric-Level Agreement With Human Graders Is Strong in Rat OIR Model and Mixed in Mouse OIR Model

We subsequently compared performance of our deep learning model to our human graders. For metric-level analysis, we averaged percent AVA and percent IVNV across the three human graders to obtain a single value for each image and then compared these values with the corresponding values generated by our deep learning model. Given that the previously published deep learning model performed poorly when applied to rat OIR images, we limited direct model-to-model comparisons to mouse OIR images, where the previously published model was developed and validated.^10^

**Figure 4.**
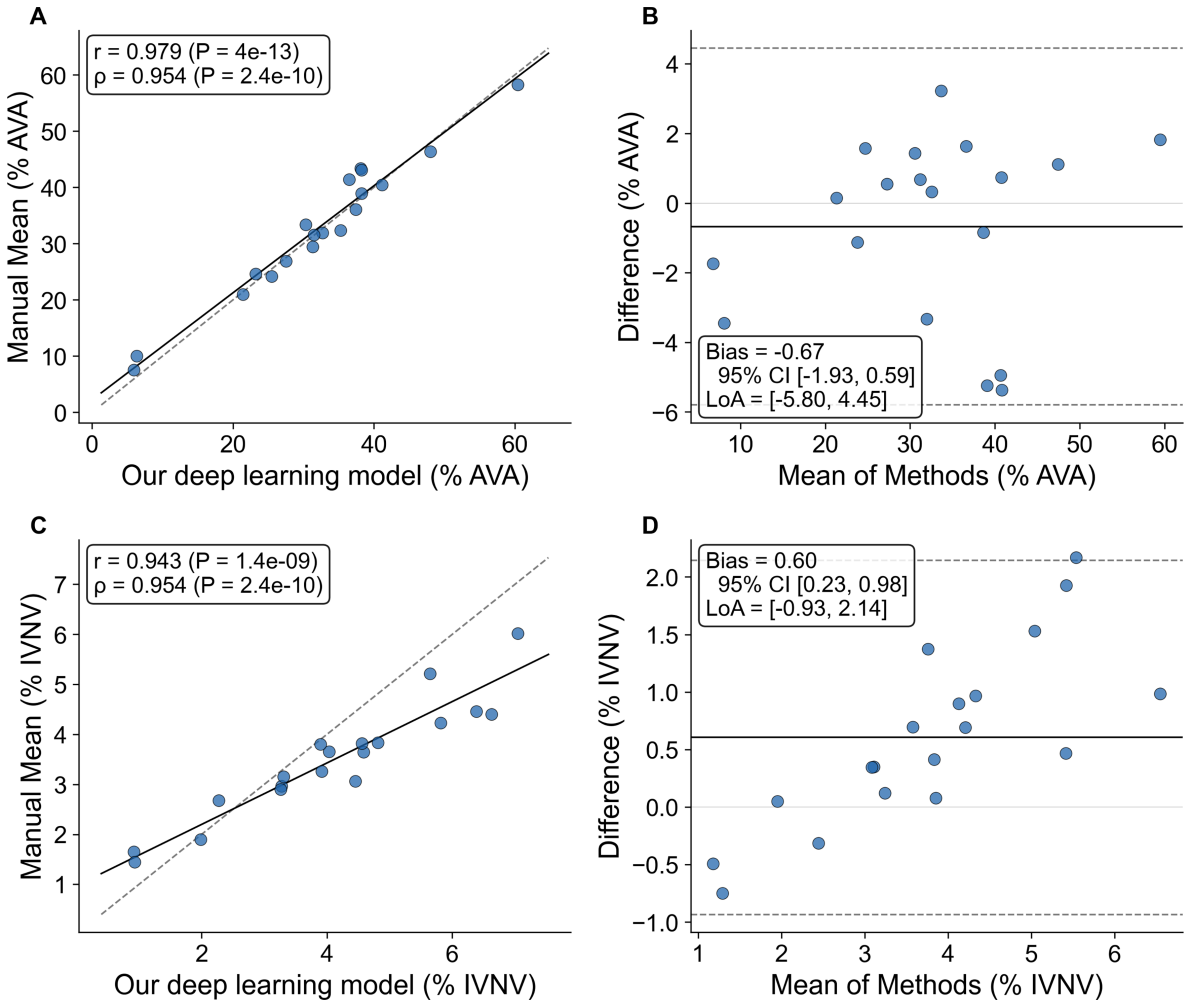
Agreement between our deep learning model and the mean of manual graders for rat percent AVA and percent IVNV. (**A**, **B**) For rat percent AVA, our deep learning model showed strong agreement with the grader mean (*r* = 0.979, *n* = 19), with a mean difference of −0.67. (**C**, **D**) For rat percent IVNV, our deep learning model also showed strong agreement with the grader mean (*r* = 0.943, *n* = 19), with a mean difference of 0.60. In scatterplots, *solid lines* indicate linear regression fits and *dashed lines* indicate the identity line. In Bland–Altman plots, the *solid horizontal line* indicates mean difference and *dashed horizontal lines* indicate the 95% limits of agreement.

For rat percent AVA, our deep learning model showed strong agreement with the mean of the human graders (*r* = 0.979, *P* = 4.01 × 10^–13^; ρ = 0.954, *P* = 2.37 × 10^–10^; *n* = 19), with a mean difference of –0.67 (95% CI, –1.93 to 0.59) and limits of agreement from –5.8 to 4.45. For rat percent IVNV, our deep learning model also showed strong correlation with the mean of the human graders (*r* = 0.943, *P* = 1.41 × 10^–9^; ρ = 0.954, *P* = 2.37 × 10^–10^; *n* = 19). The mean difference was 0.6 (95% CI, 0.23–0.98), with limits of agreement from –0.93% to 2.14% (Fig. 4). These findings support the notion that a deep learning model is able to reliably quantify percent AVA and IVNV in rat OIR retinal flatmounts.

**Figure 5.**
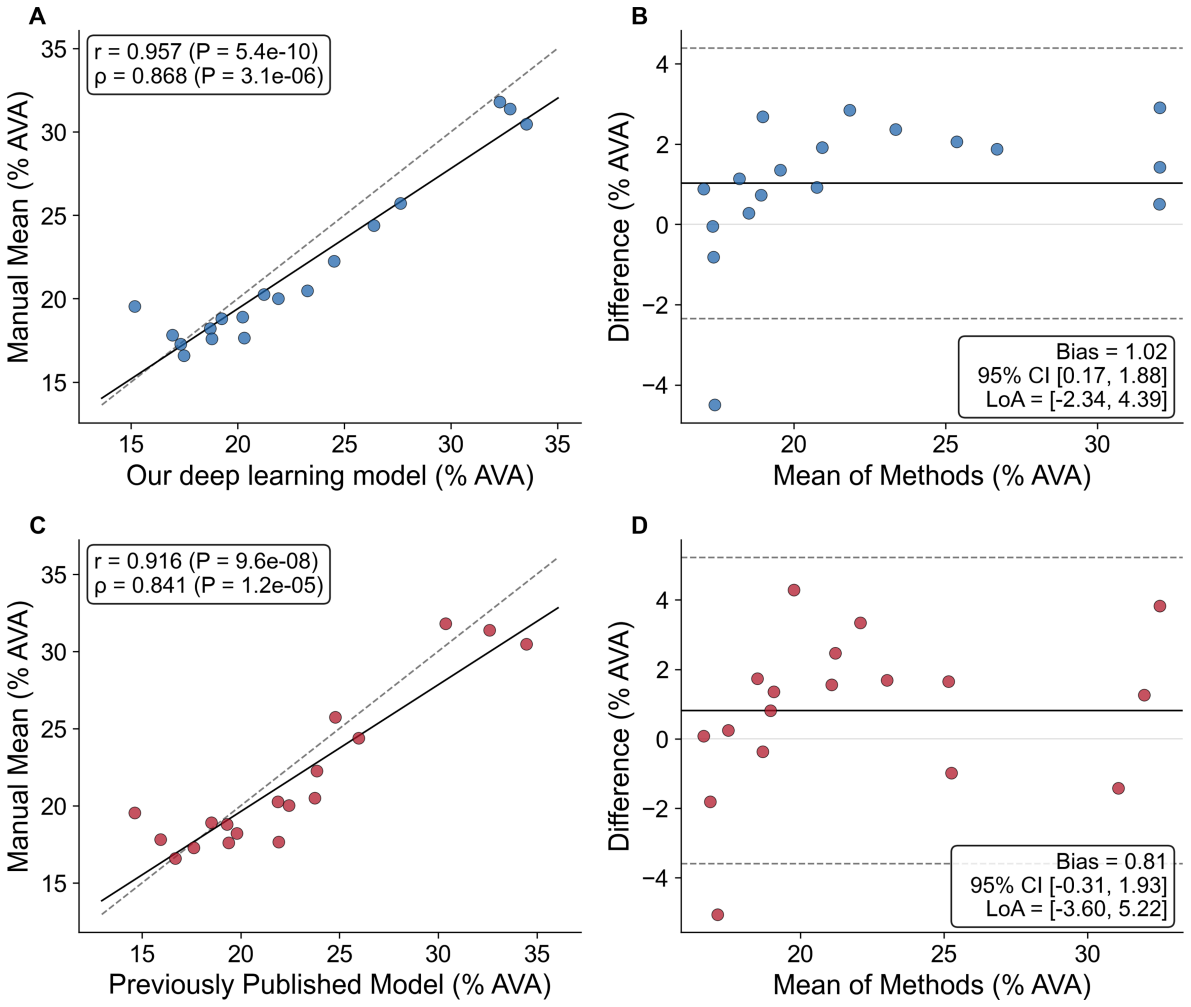
Agreement between deep learning models and the mean of manual graders for mouse percent AVA. (**A**, **B**) Our deep learning model showed strong agreement with the grader mean (*r* = 0.957, *n* = 18), with a mean difference of 1.02. (**C**, **D**) The previously published model also showed strong agreement with the grader mean (*r* = 0.916, *n* = 18), with a mean difference of 0.81. In scatterplots, *solid lines* indicate linear regression fits and *dashed lines* indicate the identity line. In Bland–Altman plots, the *solid horizontal line* indicates mean difference and *dashed horizontal lines* indicate the 95% limits of agreement.

For mouse percent AVA, both our and the previously published deep learning models showed strong agreement with the mean of our human graders. Our deep learning model achieved *r* = 0.957 (*P* = 5.39 × 10^–10^) and ρ = 0.868 (*P* = 3.05 × 10^–6^), with a mean difference of 1.02 (95% CI, 0.17–1.88) and limits of agreement from –2.34% to 4.39%. The previously published deep learning model achieved *r* = 0.916 (*P* = 9.65 × 10^–8^) and ρ = 0.841 (*P* = 1.23 × 10^–5^), with a mean difference of 0.81 (95% CI, –0.31 to 1.93) and limits of agreement from –3.6% to 5.22% (Fig. 5).

**Figure 6.**
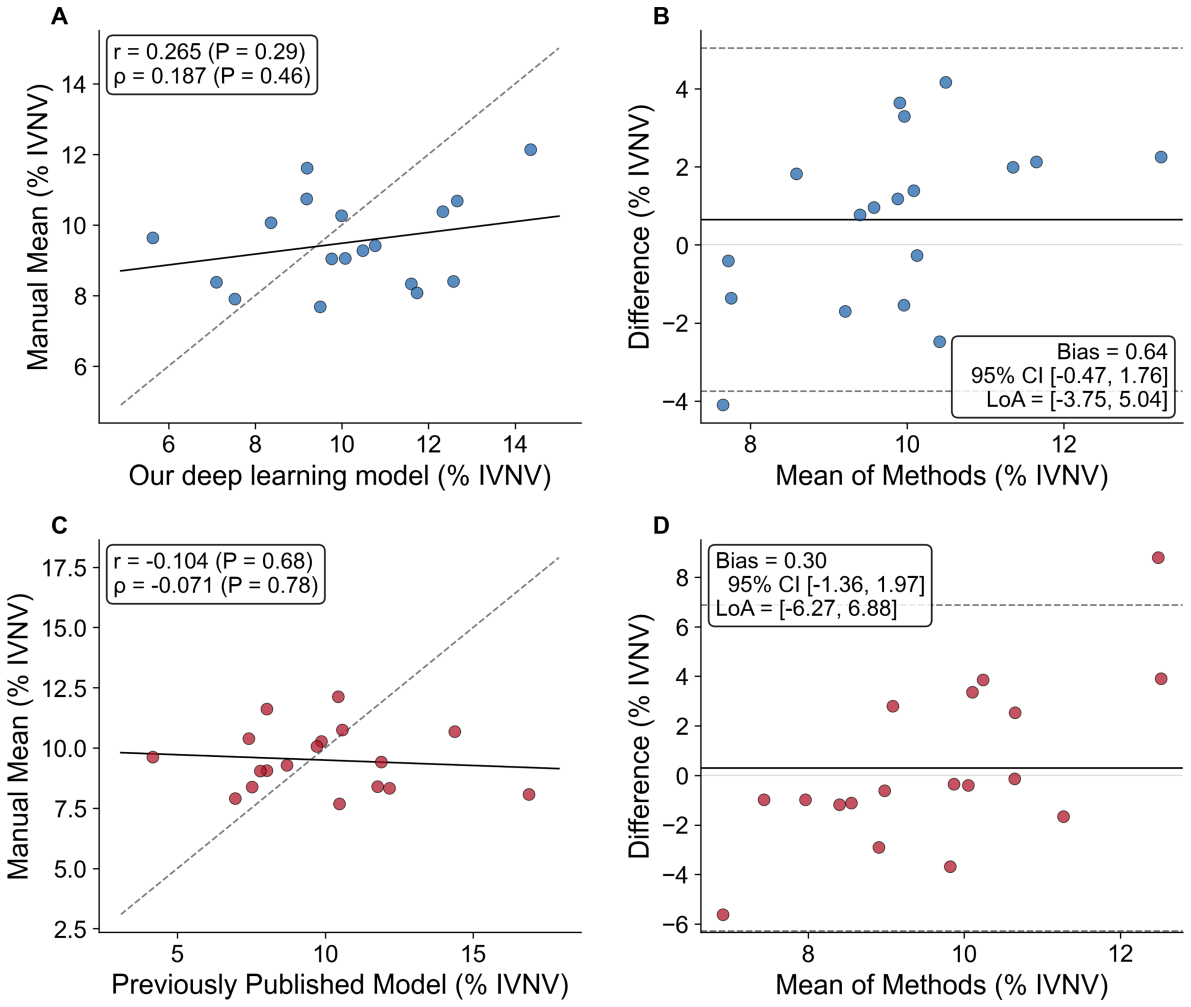
Agreement between deep learning models and the mean of manual graders for mouse percent IVNV. (**A**, **B**) Our deep learning model showed weak agreement with the grader mean (*r* = 0.265, *n* = 18), with a mean difference of 0.64. (**C**, **D**) The previously published model did not show agreement with the grader mean (*r* = −0.104, *n* = 18), with a mean difference of 0.30. In scatterplots, *solid lines* indicate linear regression fits and *dashed lines* indicate the identity line. In Bland–Altman plots, the *solid horizontal line* indicates mean difference and *dashed horizontal lines* indicate the 95% limits of agreement.

For mouse percent IVNV, agreement was lower, consistent with poor human intergrader reliability, with ICC(2,1) = –0.082 (*n* = 18). Our deep learning model showed weak correlation with the mean of the human graders (*r* = 0.265, *P* = 0.288; ρ = 0.187, *P* = 0.458; *n* = 18), with a mean difference of 0.64 (95% CI, –0.47 to 1.76) and limits of agreement from –3.75% to 5.04%. In contrast, the previously published model did not show agreement with the mean of the human graders (*r* = –0.104, *P* = 0.68; ρ = –0.071, *P* = 0.78; *n* = 18), with a mean difference of 0.30 (95% CI, –1.36 to 1.97) and limits of agreement from –6.27% to 6.88% (Fig. 6). Taken together, our findings support our hypothesis that a deep learning model is able to quantify percent AVA and IVNV in retinal flatmounts from mouse and rat OIR models.

### Pixel-Level Agreement Is High for TR and AVA and Moderate for IVNV in Rat OIR Retinal Flatmounts

**Figure 7.**
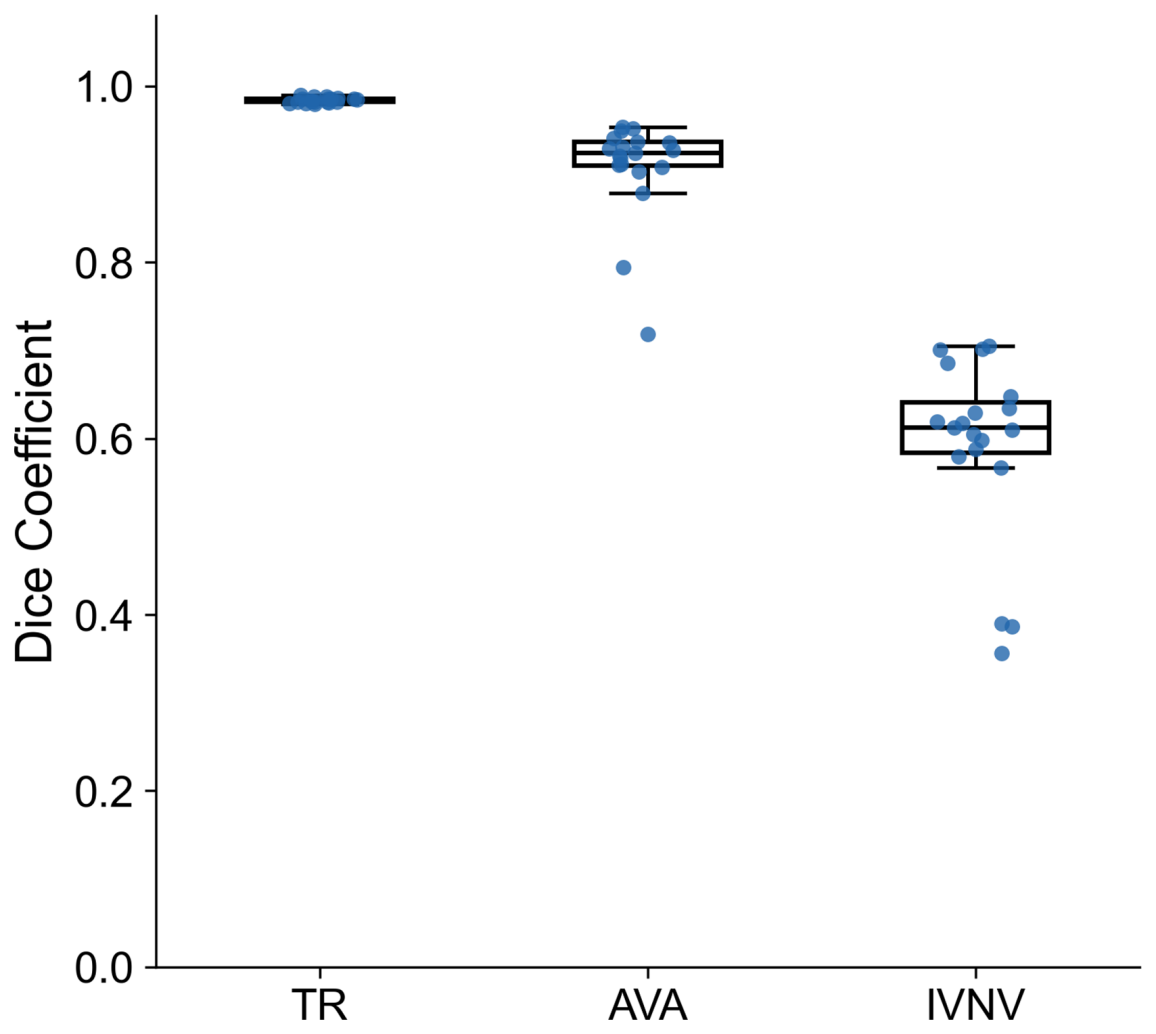
Dice scores for annotation masks generated by our deep learning model versus human consensus in rat OIR retinal flatmounts. Boxplots with individual data points show Dice coefficients for TR (median = 0.983, *n* = 19), AVA (median = 0.924, *n* = 19), and IVNV (median = 0.612, *n* = 19). The consensus reference was defined as agreement by at least two of three graders.

In addition to the metric-level analysis, we performed a pixel-level analysis to evaluate regional agreement on retinal flatmounts from the rat OIR model. We compared annotation masks generated by our deep learning model with the consensus masks from the human graders. Agreement was high for TR (median Dice = 0.983; IQR = 0.982–0.986; *n* = 19) and AVA (median Dice = 0.924; IQR = 0.910–0.936; *n* = 19), but lower for IVNV (median Dice = 0.612; IQR = 0.584–0.641; *n* = 19) (Fig. 7). These results in rat OIR support the primary component of our hypothesis that a deep learning model can be extended to automate quantification of rat retinal flatmounts.

### Mouse Annotation Mask Agreement Is Maintained Relative to the Previously Published Model

**Figure 8.**
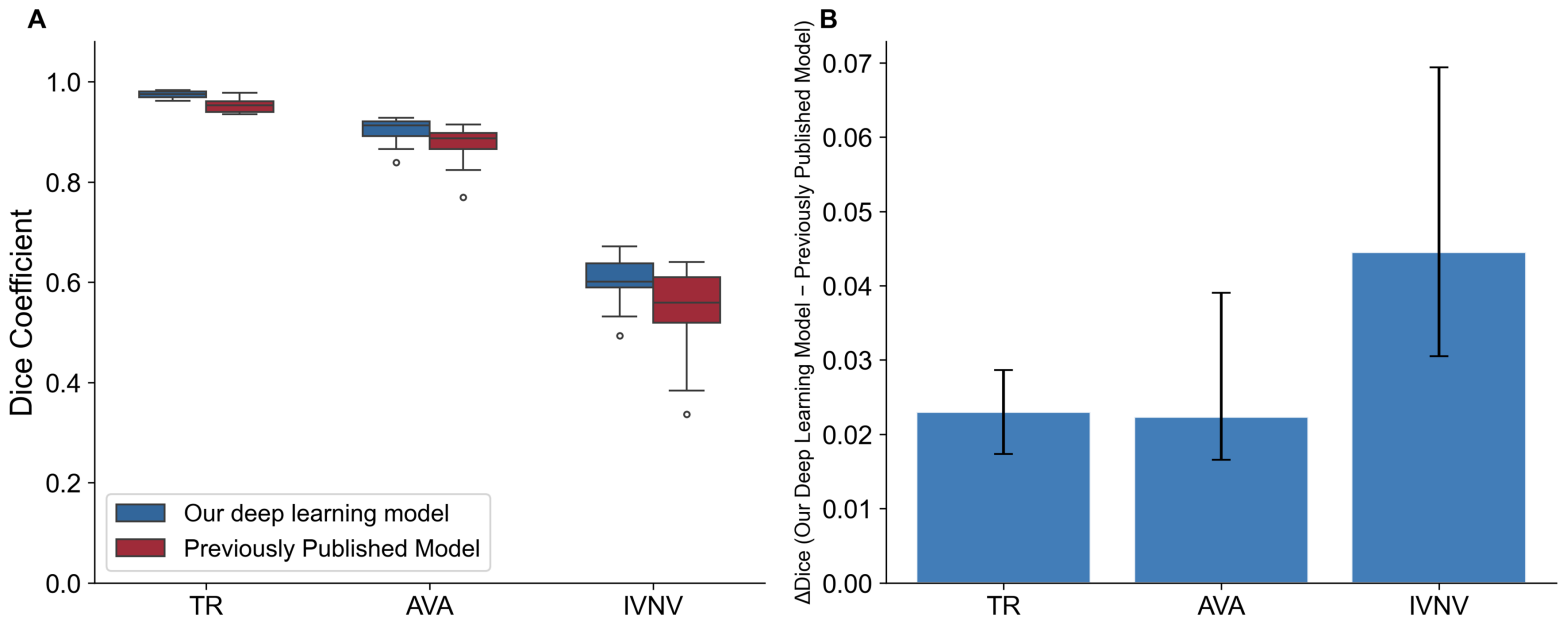
Dice scores for annotation masks generated by our deep learning model and a previously published model versus human consensus in mouse OIR retinal flatmounts, with paired per-image improvement of our model relative to the previous model. (**A**) Dice coefficients for TR, AVA, and IVNV against the human consensus masks. For our deep learning model, the median Dice coefficients were 0.975 for TR, 0.912 for AVA, and 0.601 for IVNV (*n* = 18). For the previously published model, the median Dice coefficients were 0.953 for TR, 0.887 for AVA, and 0.559 for IVNV (*n* = 18). (**B**) Paired per-image improvement in Dice coefficients for our deep learning model relative to the previously published model, with median ∆Dice values of +0.023 for TR, +0.022 for AVA, and +0.045 for IVNV. Median ∆IoU values were 0.043 for TR, 0.037 for AVA, and 0.044 for IVNV. The consensus reference was defined as agreement by at least two of three graders.

We also performed a pixel-level analysis to determine how closely the annotation masks generated by each deep learning model matched the human consensus masks in retinal flatmounts from the mouse OIR model. Using the human consensus masks as the reference, our deep learning model showed high agreement for TR (median Dice = 0.975; IQR = 0.969–0.980; *n* = 18) and AVA (median Dice = 0.912; IQR = 0.892–0.920; *n* = 18), whereas agreement for IVNV was lower (median Dice = 0.601; IQR = 0.589–0.637; *n* = 18). We then evaluated the previously published deep learning model using the same human consensus masks. Agreement for TR (median Dice = 0.953; IQR = 0.940–0.961; *n* = 18) and AVA (median Dice = 0.887; IQR = 0.866–0.897; *n* = 18) was also high, but lower than that of our deep learning model. IVNV agreement was again the lowest of the three regions (median Dice = 0.559; IQR = 0.519–0.610; *n* = 18) (Fig. 8A).

We then directly compared the annotation masks generated by our deep learning model with those generated by the previously published model^10^ for the same mouse OIR flatmounts. Relative to the previously published model,^10^ our deep learning model showed higher Dice scores for TR (median ΔDice = 0.023; IQR = 0.017–0.029; 95% CI, 0.017–0.029), AVA (median ΔDice = 0.022; IQR = 0.016–0.041; 95% CI, 0.017–0.039), and IVNV (median ΔDice = 0.045; IQR = 0.028–0.071; 95% CI, 0.031–0.069). Our deep learning model also showed higher IoU (median ΔIoU: TR, 0.043; AVA, 0.037; IVNV, 0.044) (Fig. 8B).

### Secondary Analyses Support the Deep Learning Model for Mouse IVNV and Show Substantially Reduced Scoring Time

**Figure 9.**
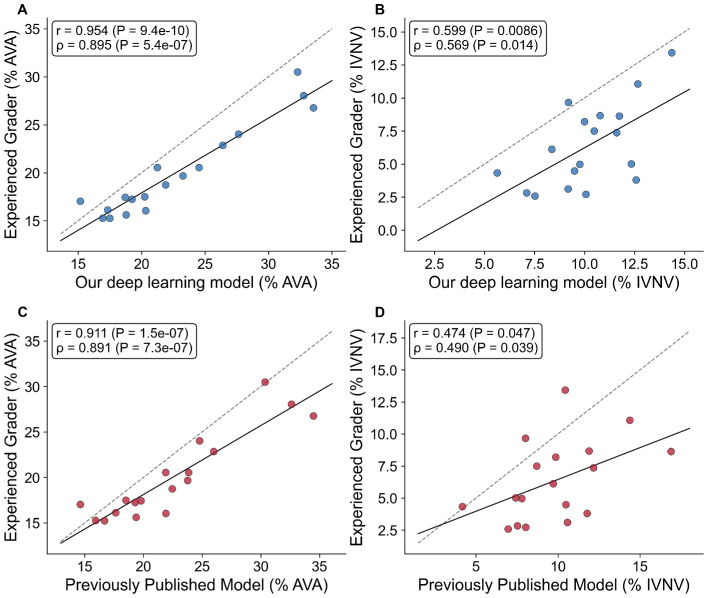
Agreement between both previous and our deep learning models and the experienced grader for mouse oxygen-induced retinopathy metrics. (**A**) For mouse percent AVA, our deep learning model showed strong agreement with the experienced grader (*n* = 18). (**B**) For mouse percent IVNV, our deep learning model showed moderate agreement with the experienced grader (*n* = 18). (**C**) The previously published model also showed strong agreement with the experienced grader for mouse percent AVA (*n* = 18). (**D**) For mouse percent IVNV, the previously published model showed weaker agreement with the experienced grader (*n* = 18). *Solid lines* indicate linear regression fits, and *dashed lines* indicate the identity line.

Although the primary analyses used consensus annotation masks and averaged metrics from all three graders, we performed secondary analyses using a single experienced grader as the reference. The most experienced grader was designated a priori for this analysis based on the assumption that their measurements were closest to the true IVNV area. For mouse percent IVNV, our deep learning model showed moderate correlation with this grader (*r* = 0.599, *P* = 0.00861; ρ = 0.569, *P* = 0.0138; *n* = 18) than the previously published model (*r* = 0.474 *P* = 0.047; ρ = 0.490, *P* = 0.039; *n* = 18) (Fig. 9). However, both models showed a positive mean difference, indicating higher percent IVNV values relative to this grader (our deep learning model: +3.8%; 95% CI, 2.54–5.05; previously published model: +3.45%; 95% CI, 1.90–5.01).

**Figure 10.**
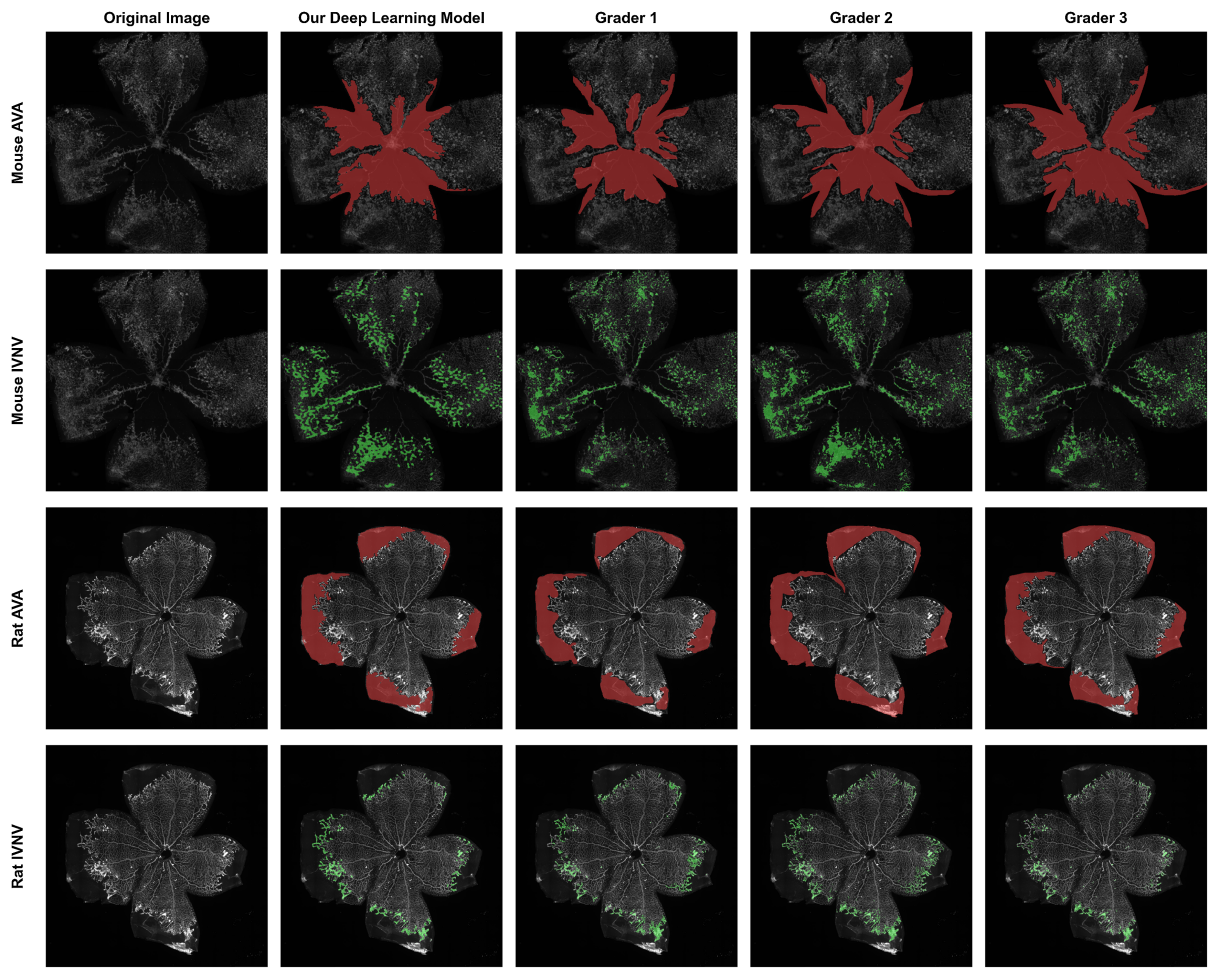
Representative annotation masks of avascular area and IVNV from our deep learning model and three human graders for mouse and rat OIR retinal flatmounts from the independent test set. Shown are representative retinal flatmounts with annotation masks (*red*, AVA; *green*, IVNV) overlaying the original image (column 1, grayscale) from our deep learning model (column 2), grader 1 (column 3), grader 2 (column 4), and grader 3 (column 5) for mouse AVA (row 1), mouse IVNV (row 2), rat AVA (row 3), and rat IVNV (row 4).

Because agreement for mouse percent IVNV appeared to vary by grader experience, we performed a separate qualitative review of IVNV annotation masks (Fig. 10). Three human experts independently evaluated masks from our deep learning model and the three human graders in a randomized, blinded four-panel layout. Across all 54 assessments (18 images × 3 reviewers), our deep learning model was selected as the most accurate IVNV mask in 45 cases (83.3%). To quantify the time required for manual grading, we also summarized annotation times by grader and species (Table).

**Table.** Times Taken to Annotate Images Between Human Graders and Our Deep Learning Model

Species	Annotator	Flatmounts, *n*	Median Time Taken Per Flatmount (min)	IQR (min)
Mouse	Grader 1	18	35.0	25.0–40.0
Mouse	Grader 2	18	40.0	35.0–45.0
Mouse	Grader 3	18	75.0	63.8–90.0
Mouse	Our deep learning model	18	0.16^*^	0.15–0.16^*^
Rat	Grader 1	19	30.0	30.0–30.0
Rat	Grader 2	19	15.0	15.0–20.0
Rat	Grader 3	19	60.0	60.0–60.0
Rat	Our deep learning model	19	0.83^*^	0.79–1.09^*^

* Model was run on an Apple M2 MacBook Pro (16-GB RAM).

## Discussion

In this study, we tested the hypothesis that a deep learning model could analyze rat retinal flatmounts while maintaining mouse performance similar to that of a previously published deep learning model trained only on mouse images. Overall, our results support the hypothesis and demonstrate that a single deep learning model can be used to quantify retinal flatmounts from both species.

We first examined consistency among our human graders. Percent AVA showed high intergrader reliability in both species, indicating that this endpoint can be measured consistently across graders. In contrast, percent IVNV showed poor intergrader reliability, particularly in mouse OIR. Although graders often identified IVNV in similar locations, they differed in the total area they annotated (Fig. 10). This variability limits the level of agreement that can be achieved between a deep learning model and a human reference for percent IVNV.

Because percent AVA and IVNV are the primary outcomes in OIR research, we evaluated model performance at this metric level. Our deep learning model showed strong correlation with human graders for percent AVA in both mouse and rat OIR, with low systematic bias. For percent IVNV in rat OIR, correlation with the mean of the human graders was also strong, but Bland–Altman analysis showed a slight upward bias (mean difference, +0.60%; 95% CI, 0.23–0.98), suggesting that our deep learning model might annotate IVNV more broadly than human graders. Although the observed bias may still allow useful relative comparisons between experimental groups, studies that require precise absolute IVNV measurement may require additional calibration to align model outputs with local grading standards.

Pixel-level comparison of annotation masks demonstrated lower spatial overlap for mouse and rat IVNV than for corresponding AVA. This likely reflects the difficulty of defining IVNV boundaries on two-dimensional flatmounts, where preretinal tufts can blend with intraretinal vasculature and borders are not always clearly demarcated. In this study, our masked human graders identified IVNV based on morphology, lectin staining pattern, and location at the avascular–vascular border. This is the gold-standard approach to quantifying rodent OIR retinal flatmount images. However, human graders are trained on retinal flatmounts and paraffin-embedded retinal cryosections labeled with ADPase staining, which can provide sharper vascular boundary definition,^26^ to help delineate IVNV from physiologic intraretinal vascularization.^27^ However, establishing a true reference standard for IVNV using fluorescently labeled flatmounts is challenging. Nonetheless, when we compared our lectin-stained mouse OIR performance with the previously published model,^10^ pixel-level analyses demonstrated that our deep learning model performed similarly to, and in some measures slightly better than, the previously published model when evaluated against human consensus annotation masks.

Given the poor intergrader reliability for percent IVNV in mouse OIR, we performed secondary analyses to better understand model performance for this endpoint. Our deep learning model showed moderate correlation with the most experienced grader (*r* = 0.599). In a blinded qualitative review, our deep learning model was selected as the most accurate IVNV annotated mask in 45 of 54 assessments by experienced reviewers. Furthermore, our deep learning model significantly reduced scoring time by up to 320-fold for mouse and 42-fold for rat. The longer processing times observed for rat images were due to their larger image size (mouse, 3800 × 3300 pixels; rat, 12,000 × 12,000 pixels). Overall, our deep learning model showed stronger agreement with the experienced grader than the previously published model, was frequently preferred over individual human annotations in blinded qualitative review, and substantially reduced scoring time. Together, these findings suggest that our deep learning model may provide a more standardized and efficient approach for IVNV annotation.

### Limitations

As with any deep learning model, our model performance is limited by data availability and quality. Our dataset is relatively small, which reduces generalizability to other labs. We attempted to minimize this limitation by including images acquired using different microscopes and from an open-source repository.^11^ However, laboratories adopting our deep learning model should validate its performance on a representative subset of their own images and, when feasible, fine-tune the model using locally annotated data. Publicly sharing locally fine-tuned models, along with their validation performance, could improve cross-laboratory generalizability and accelerate standardization in the field. Our model uses more mouse than rat OIR retinal flatmount images; however, the relatively smaller rat OIR dataset still achieved strong to moderate performance in identifying AVA and IVNV. Ground truth annotations for training data and independent tests were generated by a small number of graders, which is particularly limiting for IVNV given high levels of intergrader variability. To mitigate potential bias, we used only expert-reviewed annotations for training and ensured that the independent test set was graded by a separate group of human graders not involved in generating training annotations. However, a larger, multi-institutional panel of human graders would provide a more robust reference standard. Additionally, flatmounting can introduce tissue distortions, tears, and folds, and all images were stained using isolectin B4. The impact of these tissue artifacts on model performance was not evaluated, and model performance with alternative staining methods has yet to be studied. Finally, this model was developed exclusively on rodent OIR and is intended as a preclinical research tool; translation to human retinal imaging or clinical applications was not evaluated in this study.

## Conclusions

ROP remains a leading cause of childhood blindness worldwide, and more studies are required to understand disease mechanisms. The mouse and rat OIR models identify different features related to human ROP. The mouse model is not a representative model of delayed physiologic retinal vascularization or persistent avascular retina that occur in human infants with ROP. The mouse model offers model for genetic knockouts and high-oxygen-induced vascular damage. The rat model better recapitulates these features of persistent avascularity and delayed vascular development. These models are complementary, not interchangeable, and, although both have limitations in fully representing human ROP, together they provide the most comprehensive approach to study ROP pathophysiology.

An important consideration in OIR research is that percent AVA and IVNV may not always correlate with one another, as their relative extents depend on the underlying biological processes and signaling pathways being tested. Automated quantification tools, such as our deep learning model, can measure both endpoints independently to understand these distinct outcomes. For example, interventions that promote revascularization may reduce AVA without proportionally affecting IVNV, and anti-angiogenic therapies may reduce IVNV without promoting physiologic vessel regrowth into avascular areas. The ability to accurately quantify both outcomes across studies will facilitate more standardized evaluation.

Furthermore, the difficulty human graders experience in consistently identifying IVNV underscores the value of automated approaches but also may reflect challenges in distinguishing beneficial extension of angiogenesis peripherally versus pathologic intravitreal angiogenesis. ADPase may offer a stronger reference standard for training deep learning models in future studies. Our deep learning model may also help identify distinct morphological patterns of disordered angiogenesis. Future work could leverage model-generated IVNV masks to study IVNV morphology in greater detail. For example, self-supervised or unsupervised approaches could group IVNV regions by morphology to explore whether specific subtypes of angiogenesis exist across different experimental conditions or treatment interventions.

Additional vascular features such as capillary density are also used in some OIR studies. We did not include capillary density in our deep learning model because existing methods already quantify this feature reliably,^28^ and our primary goal was to quantify percent AVA and IVNV across mouse and rat OIR retinal flatmount images. However, our deep learning model could be extended in future work to quantify capillary density or other vascular features such as vessel tortuosity and dilation.^29^

In conclusion, our deep learning model provides an automated approach to quantify percent AVA in mouse and rat OIR and shows promising performance for percent IVNV, particularly in rat OIR. This approach has the potential to reduce manual grading burden and help standardize OIR image analysis. Additional validation across different laboratories and imaging conditions will be important.

## Supplementary Material

Supplement 1
