# Supplementary material for: Automated Deep Learning Quantification of Avascular Area and Intravitreal Neovascularization in Retinal Flatmounts of Rodent Oxygen-Induced Retinopathy Models
Source: Transl Vis Sci Technol. 2026 Jun 30;15(6):41. doi: 10.1167/tvst.15.6.41 (PMC13326888; doi:10.1167/tvst.15.6.41)
Supplement: Supplement 1 [file tvst-15-6-41_s001.pdf]

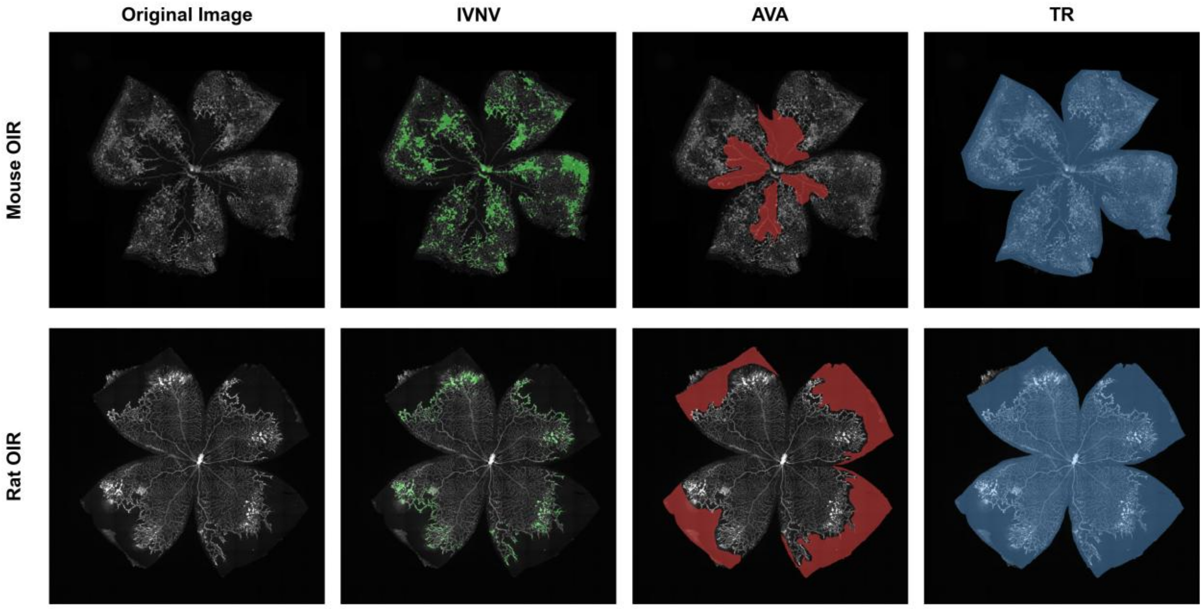

Supplemental Figure S1. Representative manual annotations from human graders in mouse and rat oxygen-induced retinopathy retinal flat mounts. Representative retinal flat mounts with annotation masks overlaying the original image (column 1, greyscale) for IVNV (column 2, green), AVA (column 3, red), and TR (column 4, blue) in mouse OIR (row 1) or rat OIR (row 2); AVA, avascular area; IVNV, intravitreal neovascularization; TR, total retina.

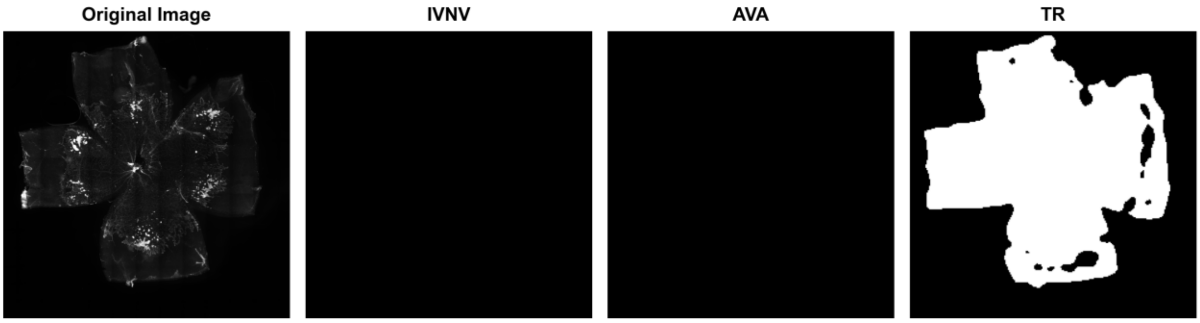

Supplemental Figure S2. Representative annotations from the previously published deep learning model when applied to a rat oxygen-induced retinopathy retinal flat mount. Representative rat retinal flat mount image with annotation masks overlaying the original image (column 1) for IVNV (column 2), AVA (column 3), or TR (column 4) by the previously published model. Blank masks in IVNV and AVA demonstrate inability to generate annotation masks by the previously published model; TR, total retina; AVA, avascular area; IVNV, intravitreal neovascularization.
